# Skin Rash as a Side Effect of Bortezomib: A Case Report

**DOI:** 10.7759/cureus.49051

**Published:** 2023-11-19

**Authors:** Marah Khaldy, Saeed Hamdan, Mohammad Amar, Munther Alshyoukhi, Hasan Arafat

**Affiliations:** 1 Internal Medicine, Augusta Victoria Hospital, Jerusalem, PSE; 2 Internal Medicine, Palestine Medical Complex, Ramallah, PSE

**Keywords:** allergy, drug side effect, multiple myeloma, skin eruption, bortezomib

## Abstract

Bortezomib is a novel proteasome inhibitor widely used in the treatment of multiple myeloma, especially in the case of recurrent, relapsing, or refractory myeloma. Its common side effects include nausea, vomiting, neuropathic pain, and hemorrhage. Cutaneous manifestations are considered rare. Here we report a case of a 72-year-old female patient, and a known case of multiple myeloma on VRD protocol (bortezomib [Velcade] combined with lenalidominde [Revlimid] and dexamethasone) after relapse. The patient presented with a complaint of raised, itchy, erythematous skin rash, starting in the soles and palms and then spreading to the whole body. A skin biopsy confirmed that the lesions were due to an allergic reaction. The patient was admitted as a case of sepsis and died. Skin rash is considered a rare side effect of bortezomib, with variable presentation and onset. The mainstay of treatment is corticosteroids.

## Introduction

Bortezomib is a relatively new proteasome inhibitor drug, and it demonstrates antiproliferative and antitumor activity by the inhibition of proteasomal degradation of several regulatory ubiquitinated proteins [1]. Bortezomib was approved for the treatment of refractory/relapsed multiple myeloma in 2003 [2], and it has demonstrated an established clinical effectiveness in mantle cell lymphoma, Waldenström's macroglobulinemia, T-cell lymphomas, and various other lymphoproliferative disorders. It has shown this efficacy both when administered as a single agent and when used in combination with other drugs [1].

Among individuals with recurrent or relapsed/refractory multiple myeloma, the use of combination therapy involving bortezomib (Velcade), lenalidomide (Revlimid), and low-dose dexamethasone (VRD protocol) has demonstrated anti-myeloma activity. This treatment approach resulted in an overall response rate of 64% and a six-month progression-free survival rate of 75% [3].

Common adverse effects of bortezomib include asthenia, nausea, vomiting, loss of appetite, thrombocytopenia, and peripheral neuropathy. Adverse cutaneous side effects concern about 10-24% of patients and have been described mainly as "rash" or "diffuse maculopapular rash." Recent literature has shown an association between the use of this medication and several severe cutaneous adverse reaction syndromes, including drug-induced hypersensitivity syndrome, toxic epidermal necrolysis, and Sweet syndrome [4-6].

In this case, we present the case of a patient with relapsed MM who was treated with bortezomib and developed skin eruption after that, with the investigation and results to diagnose.

## Case presentation

We present here the case of a 72-year-old female patient, with a past medical history of chronic renal failure (estimated glomerular filtration rate: 32, stage 3B), persistent atrial fibrillation, and multiple myeloma, diagnosed via bone marrow biopsy in October 2021. She was started on treatment with eight cycles of VTD protocol (bortezomib, 1.3 mg/m^2^ subcutaneously [SQ] on days 1, 8, 15, and 22, thalidomide 100 mg orally once daily on days 1 to 28, and dexamethasone 40 mg orally on days 1, 2, 9, 15, 16, 22, and 23). She was switched to VD protocol (bortezomib, 1.3 mg/m^2^ and SQ and dexamethasone, 40 mg PO every two weeks), as maintenance therapy. In May 2023, the patient underwent a new myeloma workup as part of her regular assessment and follow-up. New active hypermetabolic myelomatous innumerable bone and bone marrow lesions were seen on positron-emission tomography (PET) scan, and bone marrow biopsy showed 25% plasma cells. The patient was switched to VRD protocol as a new line (bortezomib: 1.3 mg/m^2^ SQ on days 1, 4, 8, and 14, lenalidomide 15 mg orally on days 1 to 14, dexamethasone 40 mg orally on days 1, 8, and 15 of the cycle) in addition to denosumab 120 mg SQ every 28 days.

**Figure 1:**
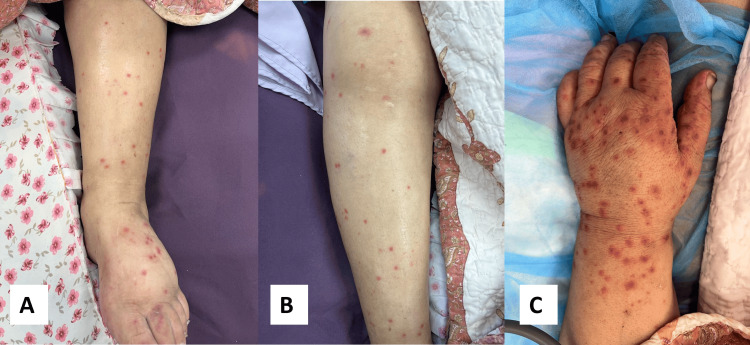
Erythematous plaques that started to appear on the patient's skin A group of erythematous plaques on our patient’s leg (A), forearm (B), and hand (C) that started to appear after receiving bortezomib. The rash started on the extremities and spread centrally.

On August 2023, the patient presented to the emergency department complaining of general weakness and perioral numbness for five days in duration. She mentioned a new skin rash, consisting of red spots, non-painful, but raised, itchy, erythematous, and edematous, which started to appear after the last dose of bortezomib. It started in the palms and soles and then continued to involve the whole body (Figure 1). On physical examination, her blood pressure was 97/58 mmHg, temperature 37.8°C, oxygen saturation 91%, and heart rate 93 beats per minute.

Laboratory investigations showed anemia, leukopenia, neutropenia, thrombocytopenia, high serum creatinine and blood urea nitrogen, hyponatremia, hypokalemia, hypocalcemia, and high C-reactive protein. The exact values are detailed in Table 1. Blood cultures grew methicillin-resistant *Staphylococcus epidermidis* (MRSE) later on.

**Table 1:** Exact values of complete blood count and basic metabolic panel of the patient Upon admission, the patient was found to have pancytopenia and acute kidney injury with electrolyte derangement. She also had an elevated C-reactive protein.

Parameter	Value	Normal Range
Hemoglobin	10 g/dL	Male: 13.8-17.2 g/dL, female: 12.1-15.1 g/dL
White Blood Cells	1.6 x 10^9^/L	4.5-11.0 x 10^9^/L
Absolute Neutrophil Count	1,200	2,500-6,000
Eosinophil Count	100	30-350
Lymphocyte Count	200	1,000-4,800
Platelet Count	19,000	150,000-450,000
Serum Creatinine	2.6 mg/dL	Male: 0.7-1.3 mg/dL, female: 0.6-1.1 mg/dL
Blood Urea Nitrogen	60 mmol/L	6-24 mg/dL
Serum Sodium	133 mEq/L	135-145 mEq/L
Serum Potassium	3.4 mEq/L	3.5-5.2 mEq/L
Serum Calcium	6.2 mg/dL	8.6-10.3 mg/dL
C-Reactive Protein	74 mg/L	0.3-1.0 mg/dL

**Figure 2:**
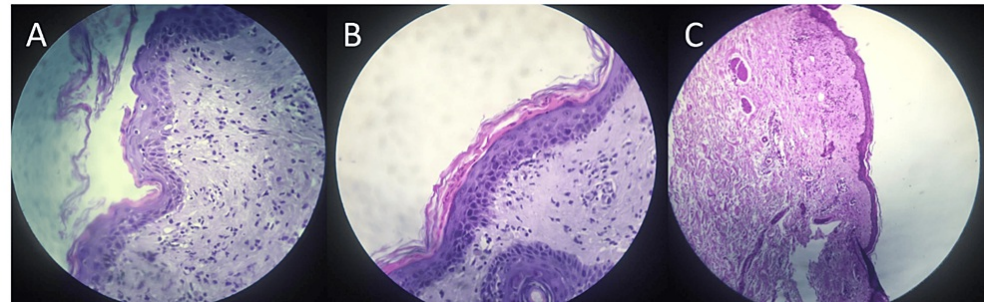
Histopathological examination of the skin biopsy Section A shows vacuolar alterations of the basal layer, section B shows parakeratosis and necrotic keratinocytes, and section C shows perivascular lymphocytic infiltrate in the upper dermis.

The patient was admitted as a case of septic shock, initially responsive to intravenous (IV) fluid resuscitation and IV antibiotics (a regimen consisting of teicoplanin and ceftazidime). She was also started on IV calcium gluconate infusion due to symptomatic hypocalcemia. A review of medications showed that the patient was on calcium carbonate, prophylactic apixaban, esomeprazole, and bisoprolol. Differential diagnoses included acute febrile neutrophilic dermatosis (Sweet syndrome), acute urticaria, allergic contact dermatitis, and drug-induced eruption. Skin biopsy was obtained, histopathology showed epidermal multifocal parakeratosis, many necrotic keratinocytes, vacuolar alteration of the basal layer, and patchy lichenoid inflammatory cell infiltrate were seen, and the upper dermis showed perivascular lymphocytic infiltrate, features in keeping with fixed drug eruption; there was no evidence of skin myeloma, dysplasia, or malignancy. Histopathology is shown in Figure 2. The patient was started on corticosteroids since admission, but its effect on skin lesions could not be assessed as the patient's condition deteriorated within days and she died.

## Discussion

Bortezomib-related cutaneous side effect is quite common, and it has been described in 15% of patients who received bortezomib as a part of their treatment [7]. Pro-inflammatory cytokines (including interleukin-6 and tumor necrosis factor-α) were believed to play a major role in the appearance of cutaneous reactions after receiving bortezomib by enhancing cell-mediated immune response [8]. It was also believed that the appearance of rash is considered a good marker for patients of non-Hodgkin's lymphoma treated with bortezomib [9]. Bortezomib-induced skin rash has been reported more frequently in patients receiving it twice weekly when compared to those receiving it once weekly [10].

The clinical manifestation of bortezomib-induced skin rash has been variable. Some cases described the lesions as erythematous and edematous plaques, other cases presented as folliculitis-like rash, while others were like eruptions that were covered with a black scar on the nose. Most of the skin rashes started to appear on the second cycle of treatment; one reported a case of skin rash that started to appear four months after the first exposure. A review of the literature showed that most of the cases respond well to systemic corticosteroid therapy and it is rarely needed to discontinue the medication [8], and the use of antihistamines was ineffective in most of the cases [11]. Our patient's skin eruption consisted of raised, itchy skin lesions. Histopathology confirmed the underlying cause of the rash, not to be confused with petechia secondary to thrombocytopenia.

Diagnosis of bortezomib-induced skin rash in our case was challenging, and this was attributed to the long time since the first exposure to this medication. As we mentioned previously, the rash appeared 21 months after receiving the first cycle and it was confirmed by histopathological findings of perivascular lymphocytic infiltration, compatible with immunological cutaneous drug eruption, with bortezomib being the only suspected culprit. A review of the literature did not reveal similar late-onset cases. Our patient was started on systemic corticoid therapy. Unfortunately, she passed away within a few days following admission, so it was difficult to assess the response of the skin rash.

## Conclusions

Bortezomib-induced skin rash has been reported in 15% of patients, with the onset being mostly following the second cycle of treatment.

In this case, we report a patient with an erythematous skin rash that appeared 21 months after the first exposure to bortezomib. It was confirmed with histopathological findings of perivascular lymphocytic infiltrations on skin biopsy. Based on that, bortezomib-induced skin rash should be suspected in patients with new-onset skin rash even if they have been on treatment for a long time. We recommend keeping it in the differential diagnosis of patients on maintenance bortezomib.
